# Watershed geomorphology modifies the sensitivity of aquatic ecosystem metabolism to temperature

**DOI:** 10.1038/s41598-019-53703-3

**Published:** 2019-11-26

**Authors:** K. J. Jankowski, D. E. Schindler

**Affiliations:** 1 School of Aquatic & Fishery Sciences, University of Washington, Seattle, WA USA; 2 US Geological Survey, Upper Midwest Environmental Sciences Center, La Crosse, WI USA

**Keywords:** Limnology, Ecosystem ecology

## Abstract

The regulation of aquatic carbon cycles by temperature is a significant uncertainty in our understanding of how watersheds will respond to climate change. Aquatic ecosystems transport substantial quantities of carbon to the atmosphere and ocean, yet we have limited understanding of how temperature modifies aquatic ecosystem metabolic processes and contributions to carbon cycles at watershed to global scales. We propose that geomorphology controls the distribution and quality of organic material that forms the metabolic base of aquatic ecosystems, thereby controlling the response of aquatic ecosystem metabolism to temperature across landscapes. Across 23 streams and four years during summer baseflow, we estimated variation in the temperature sensitivity of ecosystem respiration (R) among streams draining watersheds with different geomorphic characteristics across a boreal river basin. We found that geomorphic features imposed strong controls on temperature sensitivity; R in streams draining flat watersheds was up to six times more temperature sensitive than streams draining steeper watersheds. Further, our results show that this association between watershed geomorphology and temperature sensitivity of R was linked to the carbon quality of substrates that changed systematically across the geomorphic gradient. This suggests that geomorphology will control how carbon is transported, stored, and incorporated into river food webs as the climate warms.

## Introduction

Aquatic ecosystems play an important role in watershed carbon (C) cycles and process a substantial amount of C from the terrestrial environment^1–4^; however, there are critical uncertainties in our understanding of how aquatic ecosystem metabolism will respond to climate induced shifts in temperature and hydrology. Temperature is a primary control of organism metabolism so changes to aquatic thermal regimes will influence how C is processed through aquatic food webs. Furthermore, climate-driven changes in terrestrial productivity and litter decomposition rates will likely affect the delivery, quantity, and quality of terrestrial organic matter that supports aquatic food webs. Shifting precipitation patterns have already altered hydrological regimes^5,6^. This can further impact aquatic-terrestrial linkages through changes to dominant water sources (e.g., snow vs. rain), timing of seasonal flows^6^, soil flow paths^7^, and drought or flood conditions, which all affect the delivery of terrestrial-derived organic matter to aquatic ecosystems^8^. Therefore, not only will climate-driven temperature and hydrological change influence thermal characteristics of aquatic systems, but also the quantity and quality of organic matter that fuels ecosystem metabolism and supports recipient food webs.

One expectation for how ecosystem metabolic processes respond to changing temperature has been formalized as the Metabolic Theory of Ecology^9,10^ (MTE). MTE postulates that temperature dependence of most biochemical processes, including those involved in ecosystem C cycling, is described by a universal relationship due to the consistent biochemistry involved in metabolism across organisms and ecosystems^10–12^. This relationship can be characterized by Arrhenius kinetics, in which temperature sensitivity of metabolic rates is expressed as enzyme activation energies (*Et*) according to Eq. (1):

1 $$R(T)={R}_{ref}\,\ast \,{e}^{\frac{-Et(T-{T}_{ref})}{{K}_{b}T{T}_{ref}}}$$

where *R (T)* is the rate of respiration at temperature *T* (°K). *E*_*t*_ is the activation energy of respiration in eV and describes the slope of the of *R* with temperature, i.e., the temperature dependence of respiration. *K*_*b*_ is the Boltzmann constant (8.62 × 10-5 J °K^−1^), and *R*_*ref*_ is respiration at a standard temperature (*T*_ref_).

It remains unclear whether the temperature response of important C cycling processes, such as R, is universal^13^ or whether there are meaningful departures from this value as a result of differences in resource supply^14,15^, resource quality^16,17^, nutrient availability^18–20^ or community composition^21^. For example, there may be fundamental differences in how aquatic and terrestrial ecosystem C cycles respond to warming temperatures, which poses challenges for applying terrestrial-derived relationships to the temperature scaling of R in aquatic ecosystems. A global meta-analysis showed that the temperature sensitivity of aquatic R was higher and more variable than terrestrial R^13^, possibly reflecting greater variation in the quality of organic substrates that support aquatic R. Further, a recent study of the temperature sensitivity of metabolic rates in streams across the US showed that the greater sensitivity of R to temperature than gross primary production (GPP) could decrease net ecosystem production (NEP) in streams as global temperatures increase^22^. This higher and more variable temperature response of R has been supported by several studies in aquatic ecosystems^23–27^, but reasons for this variation are not fully understood.

Aquatic R tends to be supported by C substrates that vary more widely in their bioavailability than those in terrestrial ecosystems^8,13,28–30^, which could affect not only the gross rate of R but also how it responds to changes in temperature. Theoretical and experimental work in terrestrial systems has shown that breakdown rates of recalcitrant C sources are often more sensitive to increasing temperature than the breakdown of more labile C^16,31^, a result supported by microcosm experiments in aquatic systems^27,28^. While terrestrial R is often tightly coupled to labile substrates from gross primary production, aquatic R tends to be more often fueled by terrestrial organic matter^8,29^, which varies more in its lability than the high-quality substrates produced by aquatic autotrophs^16,32^. This difference in temperature dependence of R between terrestrial and aquatic systems could reflect greater variation in the quality and timing of C sources in aquatic systems, which are typically mediated by watershed scale controls on aquatic-terrestrial connectivity^33^, hydrology^34^, and light^35–37^.

Geomorphic features of watersheds may, therefore, ultimately control the temperature dependence of aquatic ecosystem metabolism because they constrain both the thermal regime^38^ and the quantity and quality of organic matter loaded to aquatic ecosystems. Specifically, features such as watershed slope, valley form, and network structure^39^ control hydrological interaction with floodplains and hyporheic zones, in-channel storage^40^,water residence time, and thermal regimes^38^ that in turn influence carbon retention, opportunities for microbial decomposition, and connectivity with floodplain soils and wetlands^39–42^. Further, other physical features of watersheds, such as their size, have been shown to alter the production and temperature response of organic matter by autotrophs within river networks through controlling light availability^37,43,44^. Therefore, by affecting how controlling factors are arranged on the riverine landscape and affecting processing time, geomorphic features should also constrain variation in the response of R to temperature across river networks^25^.

We estimated how growing season aquatic R responded to variation in water temperature among tributaries of a boreal river basin in southwest Alaska that fall along a distinct geomorphic gradient. We analyzed daily dynamics in *in situ* dissolved oxygen concentrations with a statistical process model^45^ to estimate rates of R and its sensitivity to changes in water temperature. This approach enabled a whole-stream assessment of how R responds to temperature and avoids some of the scaling limitations imposed by mesocosm experiments, assumptions of cross-system studies, or experiments involving artificial warming, methods that have been employed in most previous assessments of the temperature response of R^24,26,27^. In addition, we also evaluated support for a model that partitioned R into a base component (*R*_*b*_) that reflects “background” C pools that do not show diel changes in availability, and a primary production-derived component^45,46^ (*R*_*p*_) that responds to hourly changes in primary production. This model allowed us to estimate an integrated temperature sensitivity (*Et*) of *R*_*b*_ and *R*_*p*_ (*E*_*b*_ and *E*_*p*_, respectively), which have been shown to reflect diel change in the quality of C substrates^45^.

We hypothesized that *E*_*t*_ would vary predictably with watershed geomorphic features such as slope, size, and elevation through their influence on primary production, accumulation and residence time of carbon in watersheds, and aquatic-terrestrial coupling as reflected in the quantity and quality of organic substrates available to support aquatic R.

## Results and Discussion

**Figure 1:**
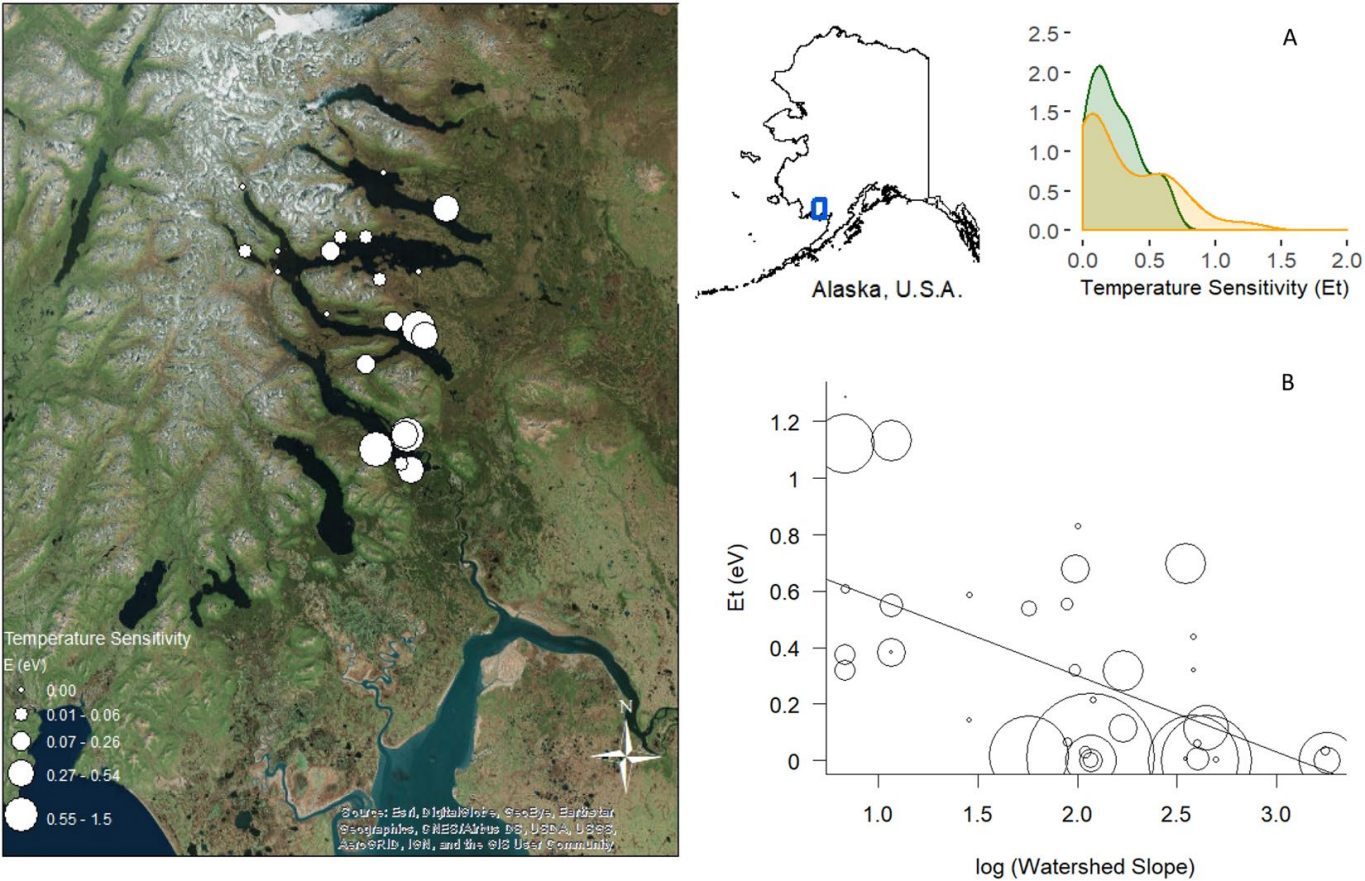
Temperature sensitivity corresponds with watershed slope. *Left panel:* Map of sites in Wood River basin. Points represent streams included in study and size of point is scaled to the magnitude of estimated temperature sensitivity (*E*_*t*_). Map of state of Alaska shows location of study area. Map was generated using the World Imagery dataset from Esri ArcMap 10.5.0.6491. Sources of satellite imagery cited on image include: “Esri, DigitalGlobe, GeoEye, Earthstar Geographic, USDA, USGS, AeroGrid, ICN, and the GIS user community”. *Right panel:* (**A**) Distribution of estimated *E*_*t*_ values - Orange represents streams included in current study and green represents streams for which we have watershed slope data in the broader Wood River Basin and could estimate an *E*_*t*_ value, (**B**) The relationship of temperature sensitivity with watershed slope (R_c_^2^ = 0.46). Size of points scale with amount of rain over the course of and one day prior to metabolism measurements.

We found wide variation in the temperature sensitivity (*E*_*t*_) of R across 23 streams and four years within the Wood River basin in southwest Alaska (Fig. 1). Posterior estimates of *E*_*t*_ values were consistently well constrained and varied between 0 and 1.94 (average = 0.33 eV, S.D = 0.33; Q_10, 5–15 °C_ ~1.7, Supplementary Tables 1 and 7). Our analyses show that a model allowing for a variable, stream-specific response of R to temperature performed substantially better in most cases than a model that set the temperature sensitivity at a single theoretical value (Supplementary Table 7). On average, R estimated at the ecosystem scale, was less sensitive to temperature than predicted by MTE^11^ and mesocosm experiments in this system^27^ (0.71 eV), but fell within the range of short-term temperature dependence for rivers estimated by other whole-stream studies (Song *et al*.^22^: median = 0.70 eV; 0.4–8.68 eV; Yvon-Durocher *et al*.^13^: median 0.53, 0.24–0.89 eV).

Simulations showed that our median posterior estimates of *E*_*b*_ were robust to changes in the range of diel temperature in the model (Supplementary Methods, Supplementary Fig. 4) and that *E*_*b*_ could be estimated independently from *k20*, the parameter governing stream reaeration rate (Supplementary Fig. 6, Supplementary Table 8). Specifically, simulations showed that posteriors of *E*_*b*_ estimates were well constrained when diel temperature variation was more than 1.0 °C, but temperature variation of less than 0.5 °C per day substantially increased uncertainty as indicated by wider posteriors. This was especially true for simulations in which we set temperature sensitivity to be low (0.32 vs 1.0 eV, Supplementary Fig. 4). However, streams in our study had average diel temperature ranges from 0.9 °C (2 streams) to 5.8 °C (mean = 2.9 °C per day), suggesting that our model was not biased under normal thermal conditions or even during storm events when diel temperature variation was reduced (1.6–1.91 °C). In addition, we evaluated whether our estimates of *E*_*b*_ were biased by or correlated with stream reaeration rates. For example, we may expect that streams draining steep watersheds have lower diel variation in temperature as a result of shorter residence time and would also have high reaeration rates, which decreases diel changes in O_2_ and, thus, could bias our estimates of *E*_*t*_ since it also depends on the daily range of those two variables. However, we found no systematic bias in the median or variation of *E*_*b*_ estimates with estimates of *k20* (Supplementary Fig. 6, Supplementary Table 8).

*E*_*t*_ was clearly associated with watershed geomorphic features across all years of the study (Fig. 1, Supplementary Fig. 5). We used a linear mixed modeling approach to quantify how aspects of watershed geomorphology influenced *E*_*t*_ and to account for among-year variation in *E*_*t*_, and found that the best geomorphic predictor of *E*_*t*_ was watershed slope (R^2^_c_ = 0.46, Fig. 1; Supplementary Table 2, Supplementary Methods). Consistent with previous experimental results in this system^27^, we found that R was considerably more sensitive to temperature in streams draining flatter watersheds than in streams draining steeper watersheds and not as responsive to other geomorphic features such as watershed area or substrate size (Supplementary Table 2). Watershed slope likely influenced *E*_*t*_ in these streams through its effects on the quantity and quality of C on the landscape^47^. In general, steep watersheds tend to accumulate little C in soils, wetlands or stream channels, while flatter watersheds accumulate C in soils and peat, within stream channels in slow-moving transient storage zones, and tend to be more hydrologically connected to carbon and nutrient sources in their floodplains^39,40,48^. There is also a tendency for R and food webs in steeper watersheds in this system to be supported more by autochthonous production of organic matter^45,48^ which is typically more labile than the terrestrially-derived organic matter loaded from flat watersheds dominated by peat bogs and deep terrestrial soils.

**Figure 2:**
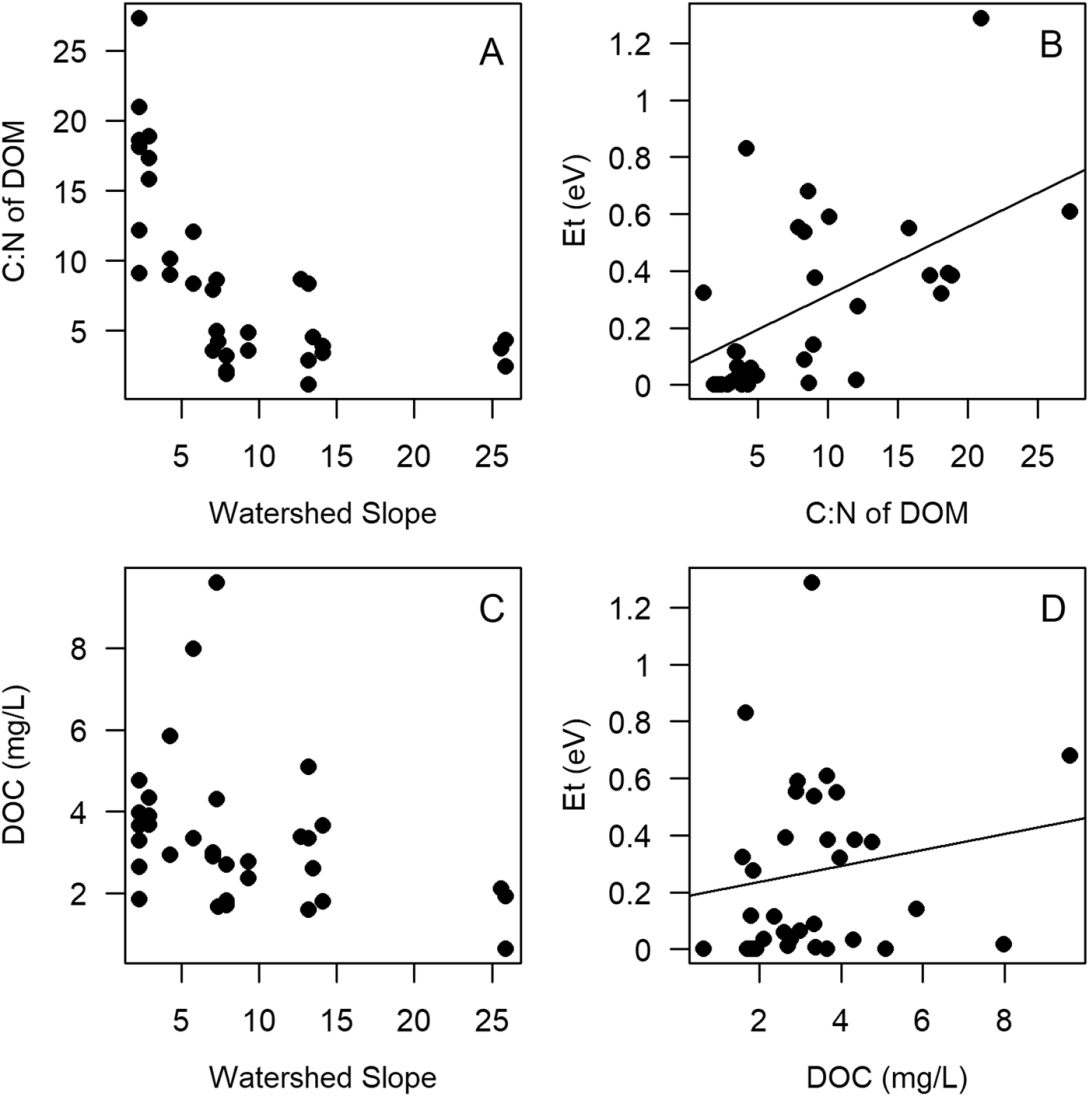
Relationship of watershed slope with (**A**) C:N ratio of DOM (R^2^_c_ = 0.69) and (**C**) total DOC (R^2^_c_ = 0.42). Relationship of temperature sensitivity (*E*_*t*_) with (**B**) C:N ratio of DOM (R^2^_c_ = 0.46) (**D**) total DOC (R^2^_c_ = 0.04).

Our results support the expectation that watershed slope controlled the characteristics of organic matter in streams. We found that DOC concentration was highest and its quality was lowest (i.e., had the highest C:N ratios) in streams draining flat watersheds (Fig. 2; Supplementary Fig. 8). Furthermore, regression models including C:N as a predictor of temperature sensitivity (*E*_*t*_) among streams were better supported by the data than models ignoring substrate quality (Supplementary Tables 3 and 4, Fig. 2). Other measures of C substrate quantity and quality (DOC, C:N, chlorophyll *a*) were also included in the best models for individual years (Supplementary Tables 3 and 4). In 2013, to supplement C:N as a metric of substrate quality, we also measured SUVA (specific ultra-violet absorbance), an index of aromatic content of organic matter^49^. SUVA was positively correlated with C:N ratio (r = 0.48, n = 11), indicating that high C:N ratios were indeed related to the presence of lower quality C substrates in streams draining flatter watersheds. Average summer temperature was also included in some of the top models, which suggests that either higher temperatures may increase decomposition rates and lead to more recalcitrant C pools or that microbes acclimated to different thermal regimes have distinct thermal responses^50^. These results suggest that although there may be some underlying universal physiological temperature response^11^, geomorphic context influences how ecosystem metabolism responds to rising temperatures through impacting C pools and stream temperature.

While watershed slope was shown to be an important control on *E*_*t*_, there remained additional unexplained variation (~50%). Several other factors could explain some of this variation, including more local scale geomorphic features that could alter residence time and deposition of organic C, seasonal succession of benthic communities, the extent of watershed forest cover and its species composition, the biomass of the organisms performing R^10,17^, and the history of hydrological events prior to the time we measured metabolism. Our results suggest that precipitation regimes affected temperature dependence of R in these streams, and that watershed geomorphology may have modified the response. Processing of organic carbon in streams and rivers varies with precipitation and hydrology patterns that influence the source, residence time, and decomposition kinetics of DOM^51^. For example, during periods of increased precipitation there would be increased hydraulic connectivity of streams with the surrounding riparian environment altering the relative amount and character of terrestrial C sources available to stream decomposers^52^ and the size of the active soil layer contributing to stream DOC fluxes^7,53^. Further, we expect that watershed and floodplain morphology mediate the degree to which streams become connected to floodplain organic matter sources during high flow events^41,54,55^. Our data suggest some of the variation in *E*_*t*_ that we observed was due to precipitation, and the degree to which this was important varied across the watershed slope gradient Specifically, we found that rainfall during metabolism measurements (plus one-day prior) decreased *E*_*t*_ in steeper streams, but may have slightly increased *E*_*t*_ in flatter watersheds (Fig. 1, Supplementary Table 5).

**Figure 3:**
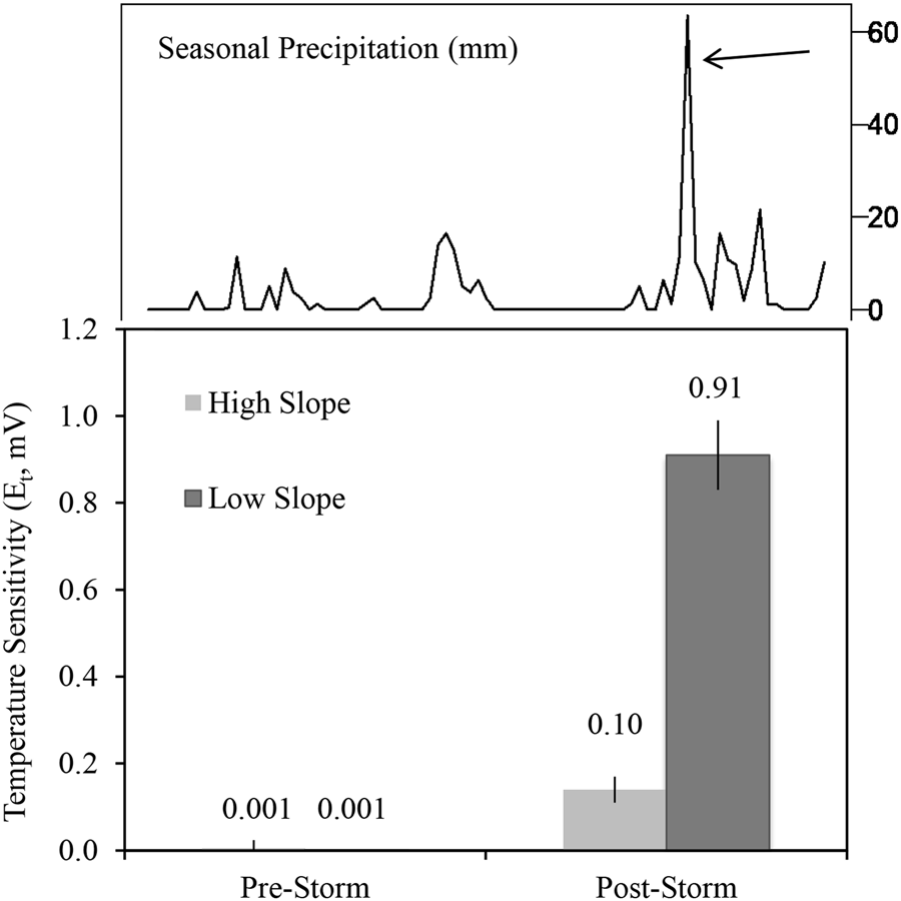
Response to precipitation. Estimates of temperature sensitivity (*E*_*t*_) before and after a large precipitation event in streams draining low and high slope watersheds in Wood River Basin. Seasonal precipitation data (May-June 2014) are shown on upper panel and arrow indicates the storm event.

One possible explanation for this is that precipitation events altered the amount and quality of organic matter in streams^51^, but to a larger degree in flatter watersheds. Work in this basin has demonstrated that DIC and food webs reflect increasing terrestrial inputs in response to storms in flatter watersheds^48^, which could increase the short-term temperature sensitivity of R, an expectation supported by our data. First, we found that rainfall prior to collection of DOC and C:N samples increased C:N slightly more in flat streams than in steep streams (Supplementary Fig. 1) and regression models suggested that an interaction between slope and precipitation for DOC and C:N was important (Supplementary Table 5). Second, we more directly evaluated the influence of a large precipitation event on temperature sensitivity, and the quantity (DOC) and quality (C:N) of C in streams draining a flat (2.6 degrees) and a steep (25.6 degrees) watershed. We found that *E*_*t*_, DOC, and C:N all increased in both streams immediately following a 64 mm rain event, but to a larger degree in the flatter stream (Fig. 3, Table 1). Taken together, these results indicate that watershed geomorphology (therefore the types and amount of organic matter on the landscape) and hydrology (the degree to which streams are connected to fringing riparian areas and hyporheic flow^56^) interact to alter carbon inputs and the sensitivity of its breakdown to temperature in streams. Whether this interaction is robust, and if it indeed reflects loading of DOM with differing lability or other factors such as bacterial community shifts, remains to be determined. Our metabolism deployments were relatively short, which may have also led to unexplained residual variation in these relationships if stream rates were highly variable within the summer base flow period during which we measured them. However, previous work in this system showed relatively consistent conditions and metabolic rates during the period over which we measured metabolism (between snow melt and prior to the arrival of spawning salmon^57^) and we observed consistent results among years for individual streams (Supplementary Fig. 5, Supplementary Table 7). In any case, further work is needed to test these hypotheses and assess the temporal and spatial scales over which these interactions occur in river networks.

**Table 1:** Precipitation event and temperature sensitivity.

Stream	Watershed Slope (degrees)	Period	*E*_*t*_ (mV)	P: R	DOC (mg L^−1^)	C: N of DOM
Elva	25.6	Pre-storm	0.001 (0.001, 0.08)	1.99	0.96 (0.32)	2.19 (0.64)
Elva	25.6	Post-storm	0.10 (0.12, 0.15)	0.79	1.12 (0.11)	2.60 (0.13)
Cham	2.3	Pre-storm	0.001 (0.001,0.001)	0.53	1.31 (0.00)	7.25 (0.12)
Cham	2.3	Post-storm	0.91 (0.88,0.94)	0.10	7.50 (0.59)	20.89 (4.0)

Table shows the effect of a large precipitation event on stream carbon substrates and temperature sensitivity. Data shown in parentheses are error values. For *E*_*t*_, error values are the width of 90% credible interval from its posterior distribution. For DOC and C:N error is reported as the standard deviation among replicate samples. C:N refers to ratio of the DOM.

Interestingly, our average estimated *E*_*t*_ values were lower than expected from mesocosm experiments in this system^27^ (Supplementary Fig. 3), but similar to values estimated in other stream ecosystems using whole-stream approaches^22^. There are several possible explanations for these differences. First, we used *in situ* diel oxygen and temperature changes rather than experimental mesocosm warming to estimate *E*_*t*_, which reflects respiration of the whole stream ecosystem. Mesocosm experiments by nature include only a portion of the benthos, whereas ecosystem metabolism measurements represent respiration occurring in the stream channel as well as in the hyporheic and fringing riparian zones. If diel variation in temperature and oxygen is lower in these subsurface areas than in the stream channel^58^, as we have seen in this system, this could lower the ecosystem-scale temperature sensitivity relative to what would be measured for surface sediments that experience larger daily swings in temperature and oxygen^59^. In addition, the magnitude and concentration of groundwater flux has been shown to influence rates of R^60^ and may also impact estimates of *Et*. Because these are gravel bed streams with shallow flow paths resuting from a clay lens that restricts deep groundwater flow (D.E. Schindler, pers. observation), we have assumed groundwater input is negligible and have not accounted for it within our modeling framework. How changes in groundwater translate into changes in temperature sensitivity is not known but is likely an additional important avenue for research. Second, it is possible that more labile C sources from autocthonous sources become limiting over the duration of mesocosm experiments, thereby increasing the temperature response of R through altering substrate quality. Few studies have used a whole ecosystem approach to estimating temperature dependence of aquatic R^23,24,36^, but the one study that used an approach similar to ours (i.e., modeling temperature sensitivity responses to local temperature fluctuation) found an even greater range of values than we observed within boreal forest streams and across biomes in their study^22^ (0.4–8.68 eV). Third, our model only accounted for diel variation in photosynthetic biomass pools (associated with *Rp*), not microbial or allochthonous biomass pools. We assumed the latter remained constant over a day (i.e., “background R”^45^), which could resulted in observed deviation from expectations of MTE^10^. In any case, this *in situ* approach produces a more accurate estimate of whole-stream temperature sensitivity than mesocosm experiments, and these differences further emphasize the need to assess and constrain these responses at this scale.

It is also important to consider how the short-term temperature response we measured here, and which has typically been measured in other aquatic ecosystem studies, translates to a longer-term response and fits into our understanding of how aquatic ecosystem metabolic rates will respond to global change. While a substantial body of work in soils and terrestrial ecosystems explores how short-term warming responses inform our understanding of long-term effects of climate warming on R, much less work exists in aquatic ecosystems^13^. Ours and other studies report that the temperature sensitivity of R is more variable among aquatic than terrestrial ecosystems^13^, which suggests that predicting long-term warming responses of aquatic metabolism may depend less on understanding their “inherent” temperature response than on understanding how dynamic biomass and resource pools that affect the temperature response respond to long-term warming trends. Further, although the temperature dependence we have estimated here represents the response to diel temperature change, the modeling framework we use here provides a means to use increasingly available metabolic data captured across seasons and years^61,62^ to understand the temperature response of aquatic R over longer time scales. Last, recent work has suggested that the temperature dependence of GPP may also be highly variable and higher than predicted by MTE, which can impact net ecosystem production and emissions as climate warms^22,44^. Here we modeled GPP as temperature invariant, but our results suggest that the temperature response of GPP may respond similarly to watershed physical features given their control on light and thermal regimes^35,63^. Further work is required to understand longer term temperature responses in aquatic systems, such as those that represent changes across seasons or years^13^, but our results take an initial step towards characterizing how the response of R to temperature may vary spatially across river networks.

In summary, our results suggest that physical features of watersheds provide a means with which to scale the thermal sensitivity of stream metabolic rates across river networks. We show that watershed geomorphology controls the basic drivers of aquatic R and its sensitivity to thermal variation (DOC, C:N, and stream temperature), which implies that basic descriptors such as watershed slope can be used to scale across more complex river basins. While our study was limited in both space (across a single river basin) and time (summer base flow), our modeling approach paired with increasingly available metabolism^62^ as well as GIS or remotely sensed data on watershed geomorphic features and vegetation can be easily expanded temporally and spatially to generate estimates of how carbon processing will respond to increasing temperatures at the watershed scale. How these relationships with geomorphology vary across seasonal changes in temperature, at longer time scales, or in developed regions where geomorphic characteristics have more subtle effects on C storage and quality^64^ remains to be quantified, but will be a fruitful area for future research. In the case of our study of a boreal river system, responses during the growing season may well be representative of most of the annual scale respiration given low temperatures and long periods of ice and snow cover.

Climate change has broad implications for thermal and hydrological regimes in stream ecosystems, and is already altering aquatic-terrestrial linkages through melting permafrost^65^, and “browning”^66,67^. Our study highlights how watershed geomorphic features control organic matter transport to aquatic ecosystems and influence how aquatic ER responds to variation in temperature. These results can help guide the development of a framework for scaling climate and land use^64^ driven changes to aquatic carbon dynamics from single streams to river basins based on simple geomorphic characteristics of landscapes.

## Methods

### Field site

This study was conducted in 23 2^nd^–4^th^ order streams of the Wood River basin (59°20’N, 158°40’W) in the Bristol Bay region of southwest Alaska during the summers of 2010–2013. The majority of the Wood River basin lies within the Wood-Tikchik State Park and contains five large, interconnected lakes fed by numerous small streams that drain through the Wood River into Bristol Bay. This region is one of the fastest warming on Earth (Maurer *et al*. 2007), and is characterized by extensive peatlands with the potential to release substantial amounts of C to the atmosphere with ongoing climate warming. The Wood River basin has considerable variation in geomorphic conditions among sub-watersheds that produces a distinct gradient in watershed slope and elevation among streams^38^ (Supplementary Table 6).

### Metabolism & temperature sensitivity

Water temperature and [O_2_] were recorded at a single station near the outflow of each stream for 3–8 days each year during the summer base flow period at 10-min intervals with a YSI 6600 V2 sonde equipped with an optical dissolved oxygen (ROx) sensor. Sensor [O_2_] measurements were calibrated in an oxygen-saturated bath, and cross-checked with [O_2_] measurements on a subset (n = 10 streams, 3 replicates per stream) as determined by Winkler titrations at the time of sonde deployment or retrieval. Irradiance at the water surface was measured directly in 10-min intervals at one of two stations no more than 30 km away with a HOBO universal weather station PAR sensor (Onset Computer Corp., Bourne, MA, USA).

Mean width and depth were measured at 10 transects over 200 meters upstream of the sensor (every ~20 m). At each transect we measured width using a laser range finder (Laser Technology Incorporated, Englewood, CO) and measured depth at five points across the stream channel. All depth measurements were averaged to generate a mean depth value for each stream. Our objective was to assess spatial variation across multiple streams during the short summer baseflow period between snow melt and the arrival of spawning salmon in late July/early August each year. Therefore, we rotated multiple sondes around 11–16 streams each year (23 unique streams in the four-year dataset), which meant that we were only able to capture a short period within this time frame with each deployment. We aimed to deploy sondes for at least three consecutive days, but in some cases storm events also interrupted our ability to estimate metabolism and estimates reflect shorter time periods. Across years, all metabolism measurements were taken within the same time frame (late June to early August) and during a similar time period for each individual stream. Further, the order and timing of these deployments were random across the watershed slope gradient, thereby removing potential bias between timing and geomorphic drivers (Supplementary Fig. 7).

We estimated the metabolic parameters and temperature sensitivity of ecosystem respiration by fitting a process model of ecosystem metabolism to daily changes in stream dissolved oxygen concentration, water temperature, and irradiance data^68^, as developed and reported by Jankowski^69^. We fit the model to these data to estimate rates of gross primary production (GPP), ecosystem respiration (ER), and gas exchange^45,68,69^. The model simulates changes in stream oxygen concentrations through estimating light-dependent oxygen production via photosynthesis, temperature-dependent oxygen consumption via respiration, and oxygen exchange between the stream and the atmosphere dependent on the gas transfer velocity and concentration gradient:

2 $$\frac{d{O}_{2}}{dt}=[k([{O}_{2,sat}]-[{O}_{2}])-R+P]/D$$

where [O_2_] is the dissolved oxygen concentration (mg m^−3^), [O_2,sat_] is the dissolved oxygen concentration at atmospheric equilibrium, R is the instantaneous respiration rate, P is the instantaneous rate of photosynthesis (both in units of mg O_2_ m^−2^ h^−1^), and D is the average depth (m). The first term in the above equation is the net effect of gas exchange, which is the gas transfer velocity, *k*, times the O_2_ concentration gradient.

To estimate temperature sensitivity of R, we modified the respiration portion of the equation based on Arrhenius kinetics and to reflect respiration derived from GPP. We used the model as developed by Jankowski^69^ and Schindler *et al*.^45^ that allows for variation in the substrate supporting heterotrophic respiration, which could influence the overall temperature sensitivity of oxygen consumption rates^27,70^. This model considers the potential for two substrate pools to support R: respiration of ambient carbon substrates or “background R” (*R*_*b*,_ Eq. 1), and respiration of a pool of labile organic matter produced via recent photosynthesis^46^ (*R*_*p*_;).

3 $${R}_{total}(t)={R}_{b}(t)+{R}_{p}(t)$$

*R*_*p*_ was modeled as an exponentially declining fraction of photosynthesis that occurred in 20 previous time steps, formulated as:

4 $${R}_{p}(T,t)={e}^{\frac{-{E}_{p}(T-{T}_{ref})}{kT{T}_{ref}}}\,\ast \,{\sum }_{i=1}^{n}P(t-i)\,\ast \,{e}^{-\beta \ast i}$$

According to this formulation we assume that carbon produced by some portion of *n* previous time steps of photosynthesis was directly consumed and respired by heterotrophs. The slope of an exponential decay function (β) describes the rate at which photosynthetically-derived organic matter is metabolized or washed out of the system. The greater the value of β, the lower the influence of photosynthetically-derived carbon on R.

*R*_*b*_ and *R*_*p*_ were considered to have different sensitivities to temperature. We set the *E* value for *R*_*p*_ at the theoretical E value for photosynthesis, *E*_*p*_ = 0.32 eV (Allen *et al*. 2005) and then estimated *E*_*b*_ for *R*_*b*_ from the data. Where the model converged on a clearly defined β, (i.e., there was strong evidence for two-sources of respiration, Supplementary Table 7) we use a model-integrated value of E, *E*_*t*_, which accounted for the proportional contribution of *R*_*p*_ and *R*_*b*_ to the overall estimate of R as follows:

5 $${E}_{t}={p}_{b}\,\ast \,{E}_{b}+{p}_{p}\,\ast \,{E}_{p}$$

where *p*_*b*_ and *p*_*p*_ (equal to 1-pb) are the proportions of *R*_*b*_ and *R*_*p*_ integrated over a 24-hour period. In the cases where the model did not converge on β, we assumed that there was no evidence for a two-stage R (*R*_*b*_ and *R*_*p*_) and estimated *E*_*t*_ with a single source model (*R*_*b*_ only, *E*_*b*_ = *E*_*t*_). We also examined whether estimating temperature sensitivity using a model that considered respiration rates of either one or two pools of carbon (daily change in respiration as a function of carbon pools^45^). This typically did not change the estimate for *E*_*b*_. When it did, however, the estimate of *E*_*b*_ increased slightly since *E*_*p*_ captured some of the temperature dependency of R. In all cases, however, we report a model-integrated value of *E*_*t*_ which was weighted according the proportional contribution from *R*_*b*_ and *R*_*p*_.

### Environmental data

To evaluate the influence of environmental characteristics on *E*_*t*_, we collected samples at the time of sonde deployment to measure stream chemical and physical conditions. Water samples for total nitrogen (TN), total phosphorus (TP), dissolved organic C (DOC), total dissolved nitrogen (TDN) and their C:N ratiowere taken from streams at the time of sonde deployment. Seasonal water temperature data used in regressions were taken from Lisi *et al*. who measured stream temperatures at the mouth of each stream at 60–90-min intervals throughout each summer in our study from June – September. Samples for the analysis of specific ultraviolet absorbance (SUVA) were taken once from streams in July 2013. To measure discharge, we either measured flow and depth at a cross-section of the stream using a Swoffer flow meter (Swoffer Instruments, Inc; Federal Way, WA) at the start and end of each deployment, where we had continuous water level loggers (Hobo U2O-001-04; Onset Computer Corp, Bourne, MA) we used stream-specific rating curves to convert water level to discharge. Watershed geomorphic characteristics for streams in this study were taken from Lisi *et al*.^38^ who demonstrated that watershed slope, elevation, stream particle size, watershed area, and lake area explained 84% of geomorphic variation among Wood River streams. Watershed slope and elevation captured the majority of this variation. However, they were correlated with one another, so they were tested individually in this study (Supplementary Table 2).

### Data analysis

We evaluated the influence of watershed geomorphic features and stream environmental conditions on the temperature sensitivity of stream respiration by regressing *E*_*t*_ estimated from the metabolism model described above against watershed geomorphic and stream chemistry variables. We tested the controls on *E*_*t*_ across all years in our dataset (2010–2013) and within each year independently (Supplementary Tables 2–4). When evaluating environmental and geomorphic controls across all years, we used a mixed effects modeling framework to account for repeated measurements in individual streams and across years by including a random effect of year and stream on the intercept as below:

6 $${{\rm{E}}}_{{\rm{t}}} \sim {{\rm{\beta }}}_{1}{{\rm{X}}}_{1}+{{\rm{\beta }}}_{2}{{\rm{X}}}_{2}\ldots +{{\rm{\beta }}}_{{\rm{n}}}{{\rm{X}}}_{{\rm{n}}}+(1|{\rm{Year}})+(1|{\rm{Stream}})+{{\rm{\varepsilon }}}_{{\rm{i}}}$$

We considered several alternative models to explain variation in E_t_ among streams in this river basin including several stream-specific environmental parameters as fixed effects (DOC, C:N, TN, TP, chlorophyll *a*, average temperature, seasonal and daily temperature range, discharge) to evaluate how these influenced the temperature sensitivity of R. Models to compare fixed effects were fit by maximum likelihood and compared with AIC_c_. For year-specific assessment of environmental controls on *E*_*t*_, we used step-wise multiple regressions and model selection by AIC_c_ to compare the influence of all of the same environmental parameters listed above on E_*t*_. We report R^2^ values for the fixed and random components of these models according to Nakagawa and Schielzeth^71^. We used a similar mixed model approach to evaluate the influence of hydrological changes on temperature sensitivity by testing the effect cumulative rainfall during the period of metabolism measurements and one day prior and its interaction with watershed slope.

We used the lme4 package^72^ to do the mixed effects analysis and all analyses were done in R (R Core Team 2014).

## Supplementary information

Supplementary Information
