# Author Correction: Watershed geomorphology modifies the sensitivity of aquatic ecosystem metabolism to temperature

**DOI:** 10.1038/s41598-020-60920-8

**Published:** 2020-02-27

**Authors:** K. J. Jankowski, D. E. Schindler

**Affiliations:** 1 School of Aquatic & Fishery Sciences, University of Washington, Seattle, WA USA; 2 US Geological Survey, Upper Midwest Environmental Sciences Center, La Crosse, WI USA

Correction to: *Scientific Reports* 10.1038/s41598-019-53703-3, published online 26 November 2019

The information in this Article is incomplete. The text in the Acknowledgements,

“Funding was provided by EPA STAR and ARCS Foundation fellowships and the US Army Corps of Engineers’ Upper Mississippi River Restoration Program to K.J Jankowski; NSF and the Harriet Bullitt Professorship to D.E. Schindler; and the UW Alaska Salmon Program.”

should read:

“Funding was provided by Environmental Protection Agency STAR and ARCS Foundation fellowships and the U.S. Army Corps of Engineers’ Upper Mississippi River Restoration Program to K.J Jankowski; National Science Foundation and the Harriet Bullitt Professorship to D.E. Schindler; and the University of Washington Alaska Salmon Program. Any use of trade, firm, or product names is for descriptive purposes only and does not imply endorsement by the U.S. Government.”

In addition, the Data availability section was omitted from the Additional Information section:

“Data availability: Data will be made available upon request to corresponding author.”

Finally, the following text should be included in the Supplemental Material:

“Any use of trade, firm, or product names is for descriptive purposes only and does not imply endorsement by the U.S. Government.”

