# Supplementary material for: Watershed geomorphology modifies the sensitivity of aquatic ecosystem metabolism to temperature
Source: Sci Rep. 2019 Nov 26;9:17619. doi: 10.1038/s41598-019-53703-3 (PMC6879538; doi:10.1038/s41598-019-53703-3)
Supplement: Supplementary file 1 — Supplementary Information [file 41598_2019_53703_MOESM1_ESM.docx]

“Watershed geomorphology modifies the sensitivity of aquatic ecosystem metabolism to temperature” - Jankowski, K.J. and D.E. Schindler

Sample Analysis

TN was determined using perchloric acid digestion followed by analysis with automated colorimetry. TP concentration was determined colorimetrically after persulfate digestion and reaction with molybdate and stannous chloride (American Public Health Association 2012). Samples for DOC and TDN analysis were filtered through a 0.7 um glass fiber filter (Whatman) acidified to pH 2 with hydrochloric acid and frozen until analysis on a TOC/TN Analyzer (Shimadzu, Kyoto, Japan). Estimates of stream periphyton biomass were determined by scrubbing six rocks per stream (the primary substrate in these streams). We quantified chlorophyll *a* content per unit rock surface area via fluorometry after extraction in methanol as described in Holtgrieve et al. (2010). Benthic organic matter content of biofilm on rocks (as ash-free dry mass by loss on ignition at 500ºC for 30 minutes) was quantified in a subset of streams. Benthic chlorophyll a and AFDM were highly correlated (r = 0.93); therefore, both terms were not included in models together and models did not differ when once was included vs. the other (Jankowski et al. 2014).

Metabolism and Temperature Sensitivity Model Assessment

As described in the main text, R_b_ and R_p_ were considered to have different sensitivities to temperature. However, we set the *E* value for R_p_ at the theoretical E value for photosynthesis, E_p_ = 0.32 eV (Allen et al. 2005) and then estimated E_b_ for R_b_ from the data. *Et*, the value used in subsequent analyses, was generated from the posteriors of *Eb, Rp*, and *Rb* and the set value of *Ep* as follows:

$$E_{t}= p_{b}*E_{b}+ p_{p}*E_{p}$$

Where, *p­_b_*, is the percent contribution of *Rb* to ER and *p_p_* is the percent contribution of *Rp* to ER. Since *Eb* was directly estimated by the model, we performed simulations to test how well the model estimated *Eb* under a variety of conditions. First, we varied the magnitude of daily changes in temperature to evaluate if there was an interaction between the daily temperature range and the magnitude of the expected *Eb*. These exercises showed that the model was able to reliably estimate *E_b_* across a broad range of parameterizations of the model, although uncertainty decreased as diel temperature variation increased (Supplementary Figure 4). Second, we evaluated the ability of the model to capture *Eb* under conditions of high gas exchange, which typically dampen the magnitude of daily changes in O_2_ making estimation of dependent parameters potentially difficult. To do so, we fed the model simulated data with a range of k values and a known *Eb* value, and then assessed the model’s ability to return the expected *Eb* value. We found that under varying levels of gas exchange, we were able to estimate *Eb* confidently (Supplementary Table 8). The median *Eb* values were nearly identical to the simulated value with normally distributed posterior distributions, except in one case in which the median value was slightly higher than expected (k = 0.28).

Model parameters were estimated in a Bayesian context (Holtgrieve et al. 2010, Schindler et al. 2017). Priors were specified on a subset of model parameters -- k_20_, E_b_, β, O_2 init_, O_2 init,σ,_ -- all of which were given a uniform distributions (a range of equally probable values). P_max_, α_PI_, and R_ref_ were estimated without prior information. Posterior probability distributions for each of the parameters were estimated though the implementation of a Markov Chain Monte Carlo (MCMC) algorithm in the software program AD Model Builder (ADMB; Fournier et al. 2012). We implemented three MCMC chains with unique starting values to be sure the chains fully explored the posterior parameter space. To test for model convergence, we used the Gelman-Rubin diagnostic which tests for within vs. among chain variance, tested the saved draws for all parameters for autocorrelation of 5% using the acf function in the CODA package of R (R Foundation), and visually examined parameter traces (Plummer et al. 2006).

The fit of models with and without estimated temperature sensitivity were assessed by using the Widely Applicable Information Criterion (WAIC, Watanabe 2010; Supplementary Table 7), a penalized likelihood criterion similar to AIC and DIC for evaluating support among competing models with Bayesian estimated parameters by MCMC. WAIC incorporates a measure of model fit and complexity similarly to these other measures, but integrates across likelihood information generated from all MCMC draws. The equation to calculate WAIC is: *elppd_WAIC_ = lppd - p_WAIC_* where *ellpd_WAIC_* is the log pointwise predictive density, or the likelihood for each data point and an estimate of how well the model fits the data. *p_WAIC_* is a penalty for the effective number of parameters in the model. The model with the lowest WAIC best explains the data. Models were assumed to perform equivalently if their WAIC scores were within 2 units of each other (Watanabe 2010, Gelman et al. 2014).

In most cases, a model with estimated temperature sensitivity fit the data either the same or better than one with E set at its theoretical value (E_b_ = 0.65eV; Supplementary Table 7, Supplementary Figure 3). In some cases, the model estimated an *Eb* value of near zero, which we interpreted as background respiration rates that were insensitive to temperature (constant R). In some cases, but not all, the streams with *E*b = 0.001 had very small daily temperature ranges (varied < 1C), thus in these cases the community was relatively unresponsive to temperature, respiration was based on substrates from photosynthesis and therefore reflected that temperature sensitivity only (Schindler et al. 2017, Allen et al. 2005), or there was not enough information in the data to generate a reliably estimate given the very small change in temperature.

REFERENCES

Allen, A. P., J. F. Gillooly, and J. H. Brown. 2005. Linking the global carbon cycle to individual metabolism. Functional Ecology **19**:202-213.

Bates, D., M. Maechler, and B. Bolker. 2014. lme4: linear mixed-effects models using Eigen and S4. <http://lme4.r-forge.r-project.org/>

Fournier, D.A., H.J. Skaug, J. Ancheta, J. Ianelli, A. Magnusson, M.N. Maunder, A. Nielsen, and J. Sibert. 2012. AD Model Builder: using automatic differentiation for statistical inference of highly parameterized complex nonlinear models. Optim. Methods Softw. 27:233-249.

Holtgrieve, G. W., D. E. Schindler, T. A. Branch, and Z. T. A'Mar. 2010. Simultaneous quantification of aquatic ecosystem metabolism and reaeration using a Bayesian statistical model of oxygen dynamics. Limnology and Oceanography **55**:1047-1063.

Jankowski, K.J., D.E. Schindler, and P.J. Lisi. Temperature sensitivity of community respiration rates in streams is associated with watershed geomorphic features. Ecology 95 (10): 2707-2714.  <http://dx.doi.org/10.1890/14-0608.1>

Plummer, M., N. Best, K. Cowles, and K. Vines. 2006. CODA: Convergence diagnosis and output analysis for MCMC. R News 6: 7–11.

R Core Team (2014). R: A language and environment for statistical computing. R Foundation for Statistical Computing, Vienna, Austria. URL <http://www.R-project.org/>.

Schindler, D.E., K. Jankowski, Z.T. A’Mar, and G.W. Holtgrieve. 2017. Two-stage metabolism inferred from diel oxygen dynamics in aquatic ecosystems. Ecosphere 8(6): 1-15.

Sinsabaugh, R.L. and J.J. Follstad Shah. 2010. Integrating resource utilization and temperature in metabolic scaling of riverine bacterial production. Ecology 91(5): 1455-1465.

Spiegelhalter, D. J., N. G. Best, B. R. Carlin, and A. Van der linde. 2002. Bayesian measures of model complexity and fit. J. R. Stat. Soc. Ser. B Stat. Method. 64: 583–639.

**Supplementary Figures & Tables**

**Supplementary Figure 1 –** Relationship of C:N of DOM with watershed slope. Size of points corresponds to cumulative rainfall 5 days prior to collection of samples.


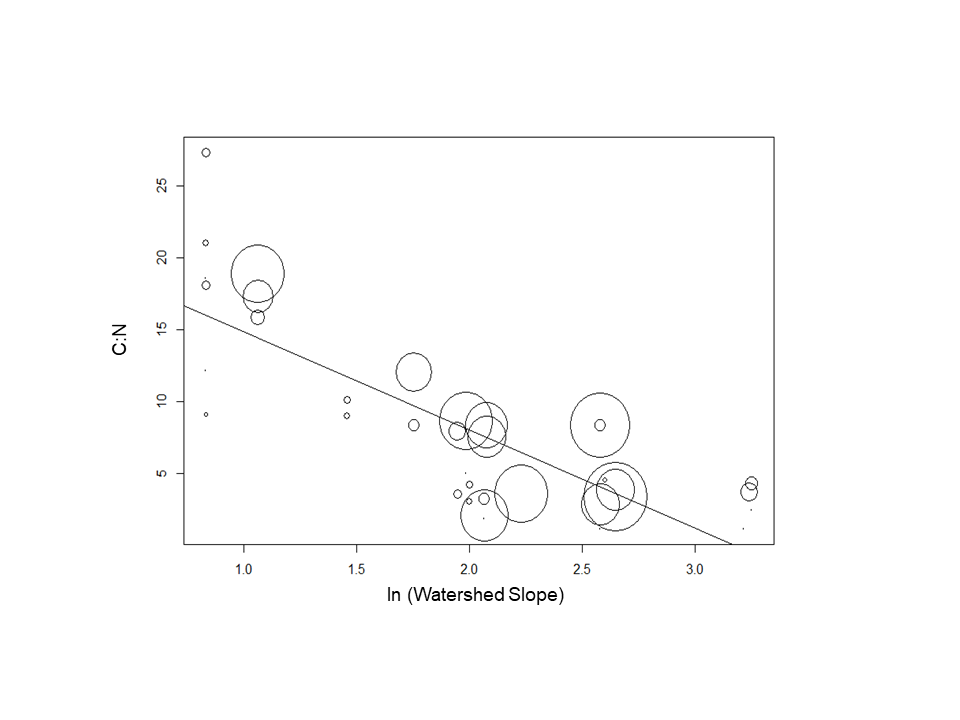


**Supplementary Figure 2.** Comparison of *E* estimated via mesocosm experiments in Wood River streams (Jankowski et al. 2014), model fitting approach in this study, and the expectation for the sensitivity of R to temperature from the Metabolic Theory of Ecology (‘MTE’; dashed line, *E* = 0.65).

**Supplementary Figure 3 –** Model fits for ecosystem metabolism shown for subset of streams (2010). Data are in black, model fit is the dashed grey line. The posterior distribution for the estimated E value for each stream is shown in bottom right of plot.

% O_2_ Saturation

Time Step

**Supplementary Figure 4.** Results of simulations that evaluated effects of diel temperature range on the model’s ability to estimate *Et*. Figure shows posterior distributions of *Et* across a spectrum of diel temperature ranges (0.5-8 C) and for a relatively small and large temperature sensitivity value (*Et*=0.32 and 1.0).

E_t_ = 0.32

E_t_ = 1.0

ΔT = 0.5°

ΔT = 1.0°

ΔT = 4.0°

ΔT = 5.5°

ΔT = 7.0°

ΔT = 8.5°

**Supplementary Figure 5.**  The relationship of temperature sensitivity with watershed slope for each year in the dataset: A) 2010: R^2^ = 0.53, n = 14; B) 2011: R^2^ = 0.74, n = 13; C) 2012: R^2^ = 0.20, n = 14; and D) 2013: R^2^ = 0.03, n = 12.

**
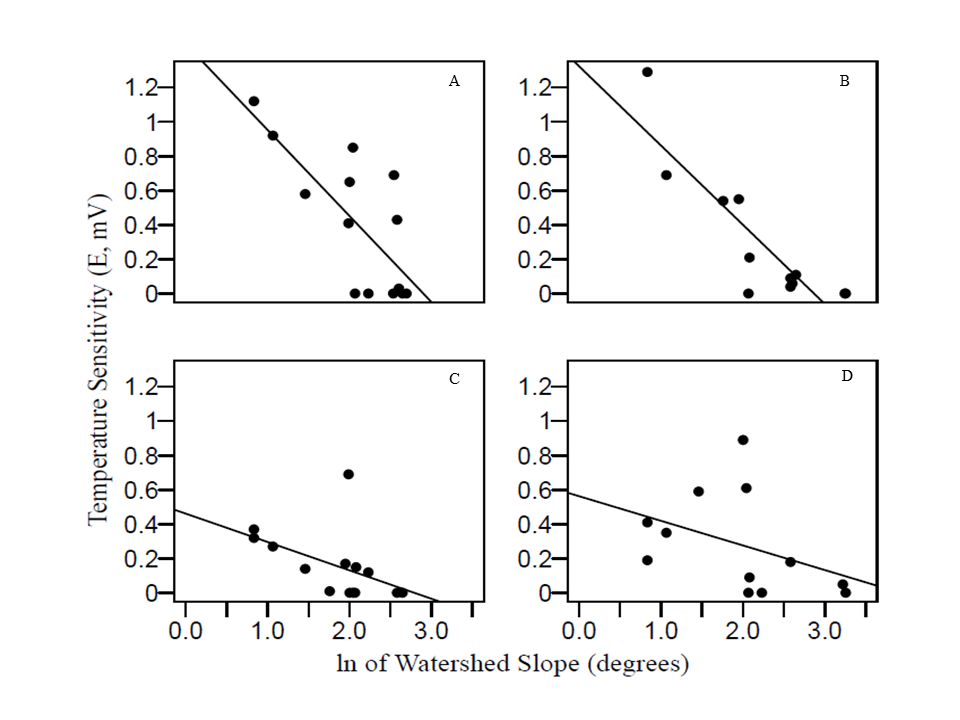
**

**Supplementary Figure 6.** Plots of posterior estimates for *k20* and *Et* for each stream in the dataset. Ellipses represent the central tendency of the posterior estimates (80% confidence intervals)


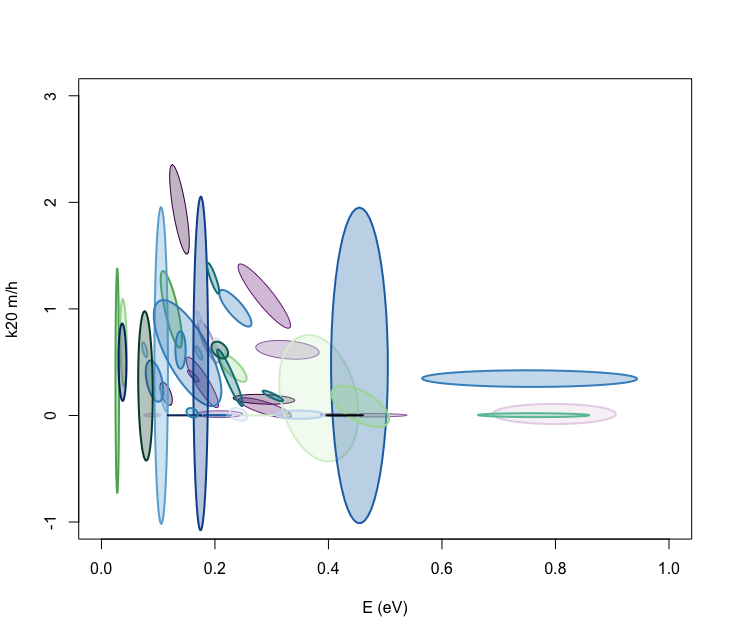


E_t_ (eV)

**Supplementary Figure 7.** Plot of sampling date with watershed slope to show that there is no bias of in the time of year streams were sampled with their slope. Line shown is regression line (p=0.98).


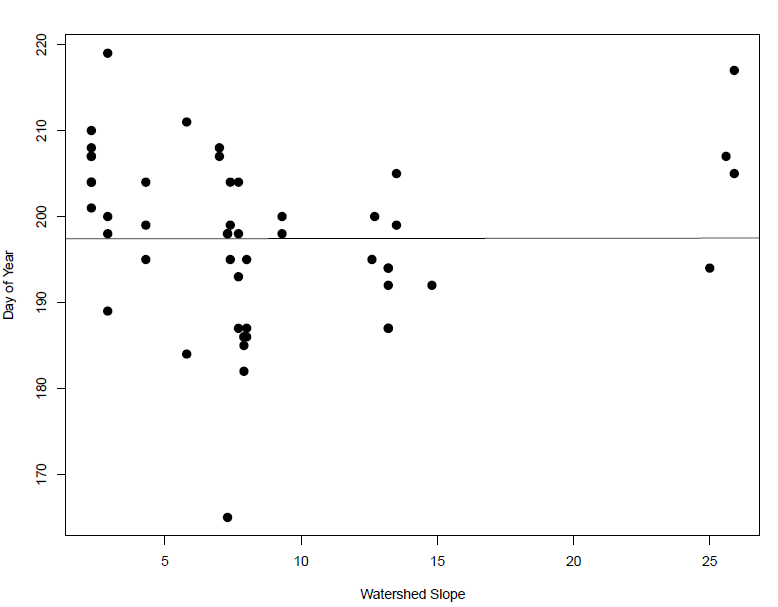


**Supplementary Figure 8.** Principal components analysis (PCA) showing covariation of geomorphic and environmental variables across streams in the Wood River Basin. DOC = dissolved organic carbon, TP = total phosphorus, TN = total nitrogen, WatSlope = watershed slope, Substrate = D84 substrate size metric, Area = watershed area, Lakes = Percent area of watershed as lakes, AvgTemp = average summer temperature, C:N = C:N ratio of dissolved pool.

**
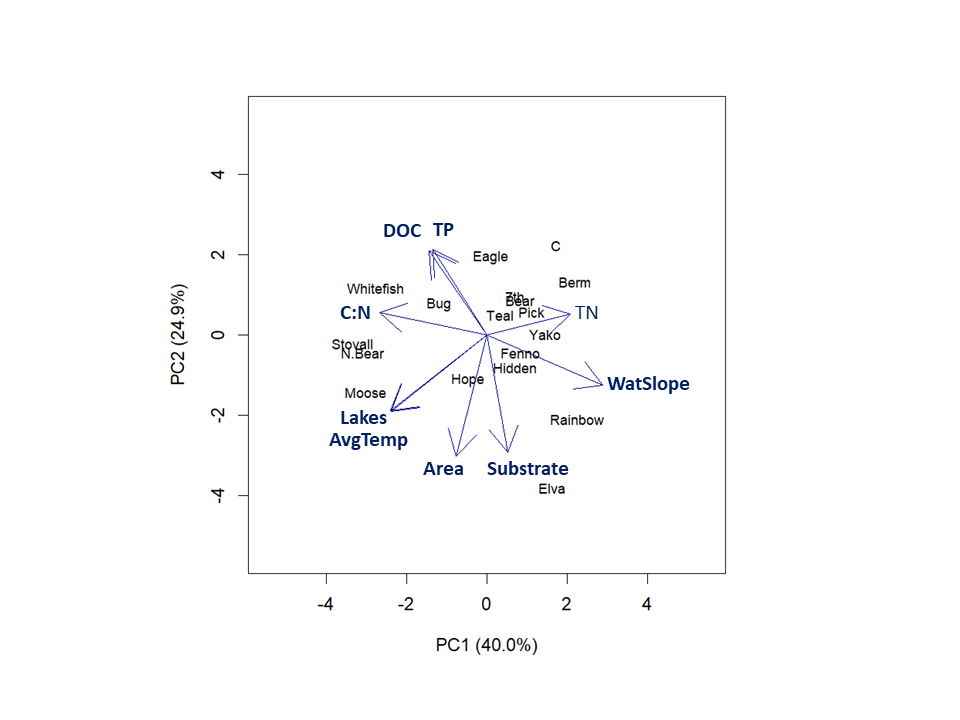
**

**Supplementary Table 1.** Average and range of estimated temperature sensitivity for four years in this study (2010-2013).

| **Year** | **Average E_t_** | **Range** | **N** |
| --- | --- | --- | --- |
| 2010 | 0.50 | 0 - 1.93 | 15 |
| 2011 | 0.34 | 0 – 1.29 | 12 |
| 2012 | 0.20 | 0 – 0.68 | 11 |
| 2013 | 0.26 | 0 – 0.83 | 11 |

**Supplementary Table 2**. Relationship of E_t_ to watershed variables: 1) watershed slope (‘slope’); 2) watershed elevation (‘elevation’); 3) watershed area (‘area’); 4) substrate size (D84; ‘substrate’); and 5) watershed contribution of lakes (%; ‘lakes’). N = number of observations, k = number of model parameters. R^2^_m_ = marginal R^2^ (variance explained by fixed effects) and R^2^_c_ = conditional R^2^ (variance explained by fixed and random effects; Nakagawa and Schielzeth 2013).

| **Model** | **N** | **k** | **AICc** | **ΔAICc** | **Likelihood** | **AICweight** | **R^2^m** | **R^2^c** |
| --- | --- | --- | --- | --- | --- | --- | --- | --- |
| E~Slope | 50 | 4 | 15.94 | 0.00 | 1.00 | 0.45 | 0.38 | 0.46 |
| E~Slope+Area | 50 | 5 | 17.60 | 1.66 | 0.44 | 0.20 | 0.39 | 0.46 |
| E~Slope+Lakes | 50 | 5 | 18.21 | 2.27 | 0.32 | 0.15 | 0.38 | 0.46 |
| E~Slope+D84 | 50 | 5 | 18.26 | 2.32 | 0.31 | 0.14 | 0.38 | 0.46 |
| E~Slope+Lakes+D84 | 50 | 6 | 20.67 | 4.73 | 0.09 | 0.04 | 0.38 | 0.46 |
| E~Slope+Area+Lakes+D84 | 50 | 7 | 22.66 | 6.72 | 0.03 | 0.02 | 0.39 | 0.46 |
| E~Area+Elevation | 50 | 5 | 27.98 | 12.03 | 0.00 | 0.00 | 0.24 | 0.27 |
| E~Elevation | 50 | 4 | 30.63 | 14.69 | 0.00 | 0.00 | 0.15 | 0.17 |
| E~Lakes | 50 | 4 | 33.33 | 17.38 | 0.00 | 0.00 | 0.10 | 0.10 |
| E~Lakes+D84 | 50 | 5 | 33.64 | 17.70 | 0.00 | 0.00 | 0.14 | 0.15 |
| E~Area+Lakes | 50 | 5 | 35.70 | 19.76 | 0.00 | 0.00 | 0.10 | 0.10 |
| E~D84 | 50 | 4 | 36.43 | 20.48 | 0.00 | 0.00 | 0.04 | 0.04 |
| E~Area+D84 | 50 | 5 | 36.92 | 20.98 | 0.00 | 0.00 | 0.08 | 0.08 |
| E~Area | 50 | 4 | 37.90 | 21.96 | 0.00 | 0.00 | 0.01 | 0.01 |

**Supplementary Table 3.** Comparison of the effects of chemical and physical variables on the temperature sensitivity of stream ER from 2011-2013. We excluded 2010 because no DOC or C:N data were available. R^2^_m_ = marginal R^2^ (variance explained by fixed effects) and R^2^_c_ = conditional R^2^ (variance explained by fixed and random effects; Nakagawa and Schielzeth 2013). Abbreviations: ‘DOC’ = dissolved organic carbon, ‘C:N’ = carbon to nitrogen ratio of dissolved organic matter, ‘AvgT’ = averaged summer stream temperature, ‘RangeT’ = range of summer stream temperature, ‘TP’ = total phosphorus, ‘TN’ = total nitrogen, ‘and Chl’ = benthic chlorophyll *a*.

| **Model** | **N** | **k** | **AIC_c_** | **ΔAIC_c_** | **Likelihood** | **AIC weight** | **R^2^m** | **R^2^c** |
| --- | --- | --- | --- | --- | --- | --- | --- | --- |
| C:N | 33 | 5 | 14.10 | 0.00 | 1.00 | 0.30 | 0.31 | 0.31 |
| C:N+AvgT | 33 | 6 | 14.40 | 0.30 | 0.86 | 0.26 | 0.36 | 0.36 |
| AvgT | 33 | 5 | 14.85 | 0.75 | 0.69 | 0.21 | 0.29 | 0.29 |
| DOC+C:N | 33 | 6 | 16.04 | 1.94 | 0.38 | 0.11 | 0.33 | 0.33 |
| DOC+C:N+AvgT | 33 | 7 | 16.91 | 2.81 | 0.25 | 0.07 | 0.37 | 0.37 |
| DOC+C:N+AvgT+TP | 33 | 8 | 20.21 | 6.11 | 0.05 | 0.01 | 0.37 | 0.37 |
| TN | 33 | 5 | 20.50 | 6.40 | 0.04 | 0.01 | 0.16 | 0.16 |
| DOC | 33 | 4 | 22.40 | 8.30 | 0.02 | 0.00 | 0.03 | 0.03 |
| RangeT | 33 | 5 | 22.83 | 8.73 | 0.01 | 0.00 | 0.10 | 0.10 |
| DOC+C:N+AvgT+TP+Chl | 33 | 9 | 22.84 | 8.74 | 0.01 | 0.00 | 0.39 | 0.39 |
| TP | 33 | 5 | 25.41 | 11.31 | 0.00 | 0.00 | 0.01 | 0.10 |
| DOC+C:N+AvgT+TN+TP+Chl | 33 | 10 | 26.48 | 12.38 | 0.00 | 0.00 | 0.40 | 0.40 |
| DOC+C:N+AvgT+RangeT+TP+TN+Chl | 33 | 11 | 30.75 | 16.65 | 0.00 | 0.00 | 0.40 | 0.40 |

**Supplementary Table 4.** Results of linear models relating stream environmental characteristics to temperature sensitivity in each year considered independently. Models from 2010 do not include DOC or C:N data because they were not available for that year. ‘DOC’ = dissolved organic carbon, ‘C:N’ = carbon to nitrogen ratio of dissolved organic matter, ‘AvgT’ = averaged summer stream temperature, ‘RangeT’ = range of summer stream temperature, ‘TP’ = total phosphorus, ‘TN’ = total nitrogen, ‘and Chl’ = benthic chlorophyll *a*.

| **2010** | **N** | **k** | **AIC_c_** | **ΔAIC_c_** | **AIC_weight_** | **R^2^** |
| --- | --- | --- | --- | --- | --- | --- |
| E~Chl | 15 | 3 | 26.4 | 0 | 0.41 | 0.35 |
| E~AvgT+Chl | 15 | 4 | 26.4 | 0.0 | 0.41 | 0.40 |
| E~AvgT+Chl+Q | 15 | 5 | 28.6 | 2.2 | 0.14 | 0.39 |
| E~AvgT+RangeT+Chl+Q | 15 | 6 | 31.5 | 5.1 | 0.03 | 0.37 |
| E~AvgT+RangeT+TN+Chl+Q | 15 | 7 | 36.2 | 9.8 | 0.00 | 0.31 |
| E~AvgT+RangeT+TP+TN+Chl+Q | 15 | 8 | 42.0 | 15.6 | 0.00 | 0.24 |
| **2011** |  |  |  |  |  |  |
| E~C:N+TN+Chl | 12 | 5 | -4.6 | 0.0 | 0.50 | 0.91 |
| E~C:N+Chl | 12 | 4 | -4.5 | 0.2 | 0.46 | 0.87 |
| E~C:N+AvgT+TN+Chl | 12 | 6 | 0.7 | 5.3 | 0.03 | 0.91 |
| E~C:N | 12 | 3 | 5.3 | 9.9 | 0.00 | 0.63 |
| E~C:N+AvgT+RangeT+TN+Chl | 12 | 7 | 9.6 | 14.2 | 0.00 | 0.91 |
| E~Chl | 12 | 3 | 14.7 | 19.3 | 0.00 | 0.19 |
| E~C:N+AvgT+RangeT+TN+Chl+Q | 12 | 8 | 24.7 | 29.4 | 0.00 | 0.92 |
| E~C:N+AvgT+RangeT+TP+TN+Chl+Q | 12 | 9 | 58.7 | 63.3 | 0.00 | 0.92 |
| E~DOC+C:N+AvgT+RangeT+TP+TN+Chl+Q | 12 | 10 | 168.4 | 173.0 | 0.00 | 0.89 |
| **2012** |  |  |  |  |  |  |
| E~C:N | 11 | 3 | -4.4 | 0 | 0.36 | 0.25 |
| E~DOC | 11 | 3 | -4.3 | 0.1 | 0.34 | 0.24 |
| E~DOC+TP | 11 | 4 | -3.5 | 0.9 | 0.23 | 0.31 |
| E~DOC+C:N+TP | 11 | 5 | 0.7 | 5.1 | 0.03 | 0.27 |
| E~DOC+C:N+TP+Chl | 11 | 6 | 6.2 | 10.6 | 0.00 | 0.18 |
| E~DOC+C:N+TP+TN+Chl | 11 | 7 | 13.6 | 18.0 | 0.00 | 0.07 |
| E~DOC+C:N+RangeT+TP+TN+Chl | 11 | 8 | 23.9 | 28.3 | 0.00 | -0.05 |
| E~DOC+C:N+RangeT+TP+TN+Chl+Q | 11 | 9 | 39.3 | 43.7 | 0.00 | -0.21 |
| E~DOC+C:N+AvgT+RangeT+TP+TN+Chl+Q | 11 | 10 | 64.9 | 69.3 | 0.00 | -0.41 |
| **2013** |  |  |  |  |  |  |
| E~C:N+AvgT+Chl | 11 | 5 | 3.2 | 0 | 0.44 | 0.64 |
| E~AvgT | 11 | 3 | 3.7 | 0.5 | 0.34 | 0.40 |
| E~C:N+AvgT | 11 | 4 | 5.3 | 2.1 | 0.15 | 0.43 |
| E~DOC | 11 | 3 | 8.1 | 4.9 | 0.04 | 0.12 |
| E~C:N+AvgT+Q+Chl | 11 | 6 | 9.5 | 6.3 | 0.02 | 0.59 |
| E~C:N | 11 | 3 | 10.5 | 7.3 | 0.01 | 0.13 |
| E~C:N+AvgT+RangeT+Q+Chl | 11 | 7 | 14.4 | 11.2 | 0.00 | 0.58 |
| E~DOC+C:N+AvgT+RangeT+Q+Chl | 11 | 8 | 27.2 | 24.0 | 0.00 | 0.51 |
| E~DOC+C:N+AvgT+RangeT+TN+Q+Chl | 11 | 9 | 48.8 | 45.6 | 0.00 | 0.41 |
| E~DOC+C:N+AvgT+RangeT+TP+TN+Q+Chl | 11 | 10 | 92.5 | 89.3 | 0.00 | 0.29 |

**Supplementary Table 5.** Results of mixed models comparing effects of watershed slope (‘Slope) and precipitation prior to metabolism measurements (“Rain”) on Temperature sensitivity (E_t_), DOC concentration (dissolved organic carbon) and C:N ratio. Includes data from 2011-2013. “Rain” for temperature sensitivity models was considered total precipitation during metabolism measurement plus one day prior. “Rain” for C models was considered two days prior to collection of the sample, which occurred on the first day of metabolism measurement. The syntax “Slope:Rain” indicates only an effect on the slope was considered, “Slope + Rain” indicates an effect on the intercept and “Slope*Rain” indicates both were considered.

**Supplementary Table 6**. Characteristics of streams in included in the study. Values for DOC (mg/L), C:N and Average Temperature (ºC) are averages across all years of the study.

| **Stream** | **Years** | **Watershed Slope (degrees)** | **Watershed Area (km^2^)** | **Watershed Elevation (m.a.s.l)** | **Substrate Size (D84)** | **Stream Width** | **Stream Depth** | **DOC mg L^-1^** | **C:N** | **Average Temperature** | **Diel Temp Range** |
| --- | --- | --- | --- | --- | --- | --- | --- | --- | --- | --- | --- |
| Chamomile | 2014 | 2.3 | 2.6 | 52 | 24 | 2.3 | 0.21 | 10.64 | 25.8 | 8.19 | 5.8 |
| N.Bear | 2011-2013 | 2.3 | 10.6 | 43 | 80 | 3.55 | 0.34 | 3.99 | 17.2 | 12.61 | 2.6 |
| Stovall | 2010- 2013 | 2.3 | 30.8 | 58 | 45 | 6.03 | 0.30 | 4.26 | 17.9 | 13.08 | 3.3 |
| Whitefish | 2011-2013 | 2.9 | 10.1 | 45 | 31 | 3.21 | 0.24 | 4.25 | 17.1 | 11.73 | 1.6 |
| Moose | 2010, 2012, 2013 | 4.3 | 94.3 | 114 | 35 | 12.26 | 0.51 | 4.40 | 9.6 | 11.97 | 3.3 |
| Bug | 2011-2012 | 5.8 | 5.6 | 64 |  | 3.44 | 0.18 | 5.87 | 10.4 | 9.82 | 4.0 |
| Teal | 2011-2012 | 7.0 | 10.6 | 100 | 48 | 4.31 | 0.21 | 5.84 | 6.7 | 9.76 | 5.5 |
| Eagle | 2010, 2012 | 7.3 | 4.1 | 80 | 46 | 3.03 | 0.20 | 8.09 | 11.5 | 7.76 | 1.5 |
| Hope | 2010, 2012, 2013 | 7.4 | 36.7 | 160 | 41 | 7.86 | 0.43 | 2.57 | 3.6 | 11.16 | 4.3 |
| Hansen | 2010, 2012, 2013 | 7.7 | 2.6 | 88 | 42 | 3.63 | 0.10 | 3.68 | 6.3 | 8.00 | 2.5 |
| Pick | 2010-2013 | 7.9 | 20.1 | 101 | 29 | 7.85 | 0.36 | 2.85 | 5.1 | 6.55 | 2.8 |
| 7th | 2010-2013 | 8.0 | 2.0 | 116 | 35 | 2.24 | 0.14 | 3.06 | 6.0 | 7.10 | 2.9 |
| Yako | 2010, 2012, 2013 | 9.3 | 13.3 | 175 | 88 | 5.36 | 0.22 | 2.58 | 4.2 | 6.39 | 1.9 |
| Yuno | 2010 | 12.6 | 17.5 | 219 | 39 | 6.06 | 0.27 | 1.78 | 5.6 | 6.12 | 3.2 |
| Bear | 2010 | 12.7 | 13.8 | 199 | 32 | 6.78 | 0.26 | 3.31 | 14.2 | 6.70 | 2.6 |
| Berm | 2011-2013 | 13.2 | 2.2 | 185 | 44 | 2.55 | 0.13 | 2.95 | 3.5 | 6.43 | 1.7 |
| Fenno | 2010-2011 | 13.2 | 38.4 | 192 | 72 | 8.06 | 0.37 | 3.77 | 8.8 | 8.38 | 3.6 |
| C | 2010-2011 | 13.5 | 1.9 | 139 | 19 | 1.84 | 0.13 | 2.60 | 4.5 | 4.30 | 0.9 |
| Hidden | 2010-2012 | 14.1 | 8.3 | 171 | 98 | 4.23 | 0.21 | 3.57 | 4.7 | 11.23 | 3.1 |
| Lynx | 2010 | 14.8 | 25.6 | 199 | 97 | 6.87 | 0.27 | 2.30 | 3.6 | 10.56 | 5.0 |
| Joe | 2013 | 25.0 | 10.8 | 414 | 115 | 5.36 | 0.19 | 0.81 | 1.1 | 7.49 | 0.9 |
| Elva | 2011 | 25.6 | 31.0 | 358 | 168 | 12.23 | 0.35 | 2.10 | 3.7 | 11.90 | 2.5 |
| Rainbow | 2011, 2013 | 25.9 | 65.6 | 493 | 80 | 15.66 | 0.40 | 1.29 | 3.4 | 5.48 | 1.2 |

**Supplementary Table 7.** Comparison of one and two-source model fits to the data for each stream included in this dataset based on WAIC. ‘N.A.’ indicates cases in which the model would not converge or parameter could not be estimated. R model = indicates whether one vs two-source model for ER was supported and used for the calculation of Et.

| **Stream** | **Year** | **Best Model** | **Eb value** | **90% Credible Interval** | **Et value** | **90% Credible Interval (Et)** | **Estimated E Model** | **Set E = 0.65 Model** | **R model** |
| --- | --- | --- | --- | --- | --- | --- | --- | --- | --- |
| 7th | 2010 | Estimated E | 1.94 | 1.54, 2.31 | 1.94 | 1.54, 2.31 | -902 | -874 | one |
| A. Bear | 2010 | E = 0.65 | 0.71 | 0.42, 1.00 | 0.69 | 0.34, 1.00 | -410.8 | -411.8 | one |
| C | 2010 | Estimated E | 0.008 | 0.001, 0.16 | 0.008 | 0.001, 0.16 | -1198 | -1192 | one |
| Eagle | 2010 | E = 0.65 | 0.06 | 0.001, 2.65 | 0.32 | 0.32, 0.32 | -205.6 | -205.1 | two |
| Fenno | 2010 | E = 0.65 | 0.44 | 0.33, 0.57 | 0.44 | 0.33, 0.55 | -712.7 | -713.2 | two |
| Hansen | 2010 | Estimated E | 0.03 | 0.001, 0.29 | 0.03 | 0.001, 0.29 | -730 | -727 | one |
| Hidden | 2010 | Estimated E | 0.001 | 0.001, 0.003 | 0.001 | 0.001, 0.003 | -703.3 | -653.3 | one |
| Hope | 2010 | E = 0.65 | 0.65 | 0.58, 0.72 | 0.65 | 0.57, 0.72 | -743.8 | -743.8 | one |
| Lynx | 2010 | Estimated E | 0.004 | 0.001, 0.04 | 0.004 | 0.001, 0.04 | -702.5 | -665.5 | one |
| Moose | 2010 | E = 0.65 | 0.61 | 0.55, 0.65 | 0.58 | 0.53, 0.68 | -871.1 | -872.1 | two |
| Pick | 2010 | Estimated E | 0.001 | 0.001, 0.002 | 0.001 | 0.001, 0.002 | -421.9 | -339 | one |
| Stovall | 2010 | Estimated E | 1.12 | 0.86, 1.4 | 1.12 | 0.86, 1.41 | -708.9 | -699.8 | one |
| Whitefish | 2010 | Estimated E | 1.13 | 0.93, 1.35 | 1.13 | 0.93, 1.35 | -958.3 | -943.7 | one |
| Yako | 2010 | Estimated E | 0.06 | 0.002, 2.4 | 0.06 | 0.002, 2.4 | -826.2 | -825.3 | one |
| Yuno | 2010 | E = 0.65 | 0.07 | 0.001, 2.5 | 0.32 | 0.001, 2.7 | -778.7 | -778.8 | two |
| 7th | 2011 | Estimated E | 0.21 | 0.09, 0.30 | 0.21 | 0.09, 0.30 | -1757.4 | -1695.02 | two |
| Bug | 2011 | E = 0.65 | 0.61 | 0.46, 0.78 | 0.54 | 0.42, 0.67 | -448 | -452 | two |
| C | 2011 | Estimated E | 0.001 | 0.001, 0.002 | 0.06 | 0.05, 0.07 | -1235.7 | -1183.7 | two |
| Elva | 2011 | Estimated E | 0.001 | 0.001, 0.002 | 0.04 | 0.03, 0.05 | -879.2 | -765.2 | two |
| Fenno | 2011 | Estimated E | 0.09 | 0.007, 0.17 | 0.09 | 0.007, 0.17 | -1449.4 | -1339.2 | one |
| Hidden | 2011 | Estimated E | 0.006 | 0.001, 0.07 | 0.12 | 0.09, 0.16 | -880.9 | -848.5 | two |
| N. Bear | 2011 | Estimated E | 1.29 | 1.15, 1.42 | 1.29 | 1.15, 1.42 | -815.9 | -776.7 | one |
| Pick | 2011 | Estimated E | 0.001 | 0.0010, 0.0011 | 0.01 | 0.001, 0.03 | -652.7 | -136.6 | two |
| Rainbow | 2011 | Estimated E | 0.0013 | 0.0010, 0.002 | 0.001 | 0.001, 0.002 | -2573.4 | -2479.5 | one |
| Stovall | 2011 | Estimated E | 0.61 | 0.46, 0.78 | 0.61 | 0.46, 0.77 | -469 | -382 | two |
| Teal | 2011 | E = 0.65 | 0.61 | 0.54, 0.68 | 0.55 | 0.49, 0.63 | -144.7 | -145.8 | two |
| Whitefish | 2011 | E = 0.65 | 0.59 | 0.09, 1.05 | 0.55 | 0.12, 0.93 | -757.2 | -759.4 | two |
| Berm | 2012 | Estimated E | 0.001 | 0.001, 0.002 | 0.001 | 0.001, 0.002 | -1388 | -1230 | one |
| Bug | 2012 | Estimated E | 0.02 | 0.001, 0.08 | 0.02 | 0.001, 0.08 | -1397 | -1159 | one |
| Eagle | 2012 | Estimated E | 0.82 | 0.70, 0.93 | 0.68 | 0.60, 0.77 | -708.2 | -704.7 | two |
| Hidden | 2012 | Estimated E | 0.002 | 0.001, 0.003 | 0.002 | 0.001, 0.003 | -1151 | -1124 | one |
| Moose | 2012 | Estimated E | 0.14 | 0.06, 0.23 | 0.14 | 0.06, 0.23 | -1720 | -1648 | one |
| N.Bear | 2012 | E = 0.65 | 0.38 | 0.13, 0.59 | 0.37 | 0.16, 0.58 | -729 | -729 | two |
| Pick | 2012 | Estimated E | 0.001 | 0.001, 0.0011 | 0.001 | 0.001, 0.0011 | -1127 | -676 | one |
| Stovall | 2012 | Estimated E | 0.32 | 0.13, 0.51 | 0.32 | 0.13, 0.51 | -443.5 | -437.7 | one |
| Teal | 2012 | Estimated E | 0.04 | 0.001, 0.32 | 0.06 | 0.02, 0.32 | -313.6 | -303.1 | two |
| Whitefish | 2012 | Estimated E | 0.42 | 0.19, 0.64 | 0.38 | 0.24, 0.54 | -676 | -669 | two |
| Yako | 2012 | Estimated E | 0.002 | 0.001, 0.004 | 0.12 | 0.08, 0.15 | -972.8 | -937.9 | two |
| A. Bear | 2013 | Estimated E | 0.001 | .001, 0.0011 | 0.005 | 0.001, 0.009 | -1828 | -813 | two |
| Berm | 2013 | E = 0.65 | 0.32 | 0.16, 0.48 | 0.32 | 0.16, 0.48 | -1178 | -1180 | one |
| Eagle | 2013 | Estimated E | 0.001 | 0.001, 0.0011 | 0.03 | 0.03, 0.04 | -1760 | -703.5 | two |
| Hope | 2013 | Estimated E | 0.89 | 0.81, 0.97 | 0.83 | 0.75, 0.92 | -1282 | -1257 | two |
| Moose | 2013 | E = 0.65 | 0.59 | 0.53, 0.64 | 0.58 | 0.53, 0.64 | -1346 | -1345 | one |
| Nbear | 2013 | Estimated E | 0.28 | 0.21, 0.34 | 0.28 | 0.21, 0.34 | -1088.7 | -887.8 | one |
| Pick | 2013 | Estimated E | 0.001 | 0.001, 0.0011 | 0.001 | 0.001, 0.0011 | -708.3 | -626.5 | one |
| Rainbow | 2013 | E = 0.65 | 0.001 | 0.001, 0.0011 | 0.001 | 0.001, 0.0011 | -1247 | -1121 | one |
| Stovall | 2013 | Estimated E | 0.4 | 0.34, 0.46 | 0.39 | 0.34, 0.44 | -1385 | -1306 | two |
| Whitefish | 2013 | Estimated E | 0.41 | 0.19, 0.64 | 0.38 | 0.24, 0.54 | -504.9 | -457.4 | two |
| Yako | 2013 | Estimated E | 0.001 | 0.001, 0.002 | 0.04 | 0.03, 0.04 | -1731 | -1381 | two |

Supplementary Table 8. Results of simulations testing the effect of increasing k20 values on model ability to accurately estimate E_t._

| **k20 value** | **Median Et**  **(10%, 90% Credible Intervals)** |
| --- | --- |
| 0.12 | 0.326  (0.32, 0.33) |
| 0.19 | 0.317  (0.30, 0.33) |
| 0.28 | 0.335  (0.32, 0.35) |
| 0.41 | 0.326  (0.31, 0.34) |
| 0.53 | 0.329  (0.32, 0.34) |
| 0.72 | 0.311  (0.30, 0.32) |
